# Composition of Organosilicate Coatings High-Temperature Breakdown Products and Their Distribution in the Weld

**DOI:** 10.3390/ma15030699

**Published:** 2022-01-18

**Authors:** Leonid Zhabrev, Dmitry Kurushkin, Igor Mushnikov, Aleksey Shamshurin, Svetlana Chuppina, Oleg Panchenko

**Affiliations:** 1 Laboratory of Lightweight Materials and Structures, Peter the Great St. Petersburg Polytechnic University, 29 Polytechnicheskaya Str., 195251 St. Petersburg, Russia; kurushkin_dv@spbstu.ru (D.K.); mushnikov_iv@spbstu.ru (I.M.); panchenko_ov@spbstu.ru (O.P.); 2 The World-Class Advanced Digital Technologies Research Center, Peter the Great St. Petersburg Polytechnic University, 29 Polytechnicheskaya Str., 195251 St. Petersburg, Russia; shamshurin_ai@spbstu.ru; 3 NMP Group LTD, Managing Company, LTD Neva Metall Posuda, 78 Lit. N Marshal Blueher Pr., 195067 St. Petersburg, Russia; tchoup@rambler.ru

**Keywords:** organosilicate coatings, high-temperature breakdown products, non-metallic inclusions, diffusible hydrogen, MAG welding

## Abstract

The construction assembly and the repair of steel constructions painted with protective coatings are often carried out using arc welding. During the welding process, the coating in the weld zone is degrading. The protective coatings breakdown products are involved in the pore and non-metallic inclusion formation in the weld, the composition and distribution study of which makes it possible to analyze the reactions occurring during the welding. In this study, welding beads were deposited on the coated sheet surface by MAG welding. The distribution of inclusions (the average diameter and the relative content) along with the porosity in different bead zones were investigated by optical and scanning electron microscopy and digital image processing, and the chemical composition of inclusions was determined using energy-dispersive X-ray spectroscopy. The amount of diffusible hydrogen in the deposited metal was estimated with the vacuum method. In this work, four organosilicate coatings grades, differing in their purpose and heat resistance, were used, and their effect on the weld was studied.

## 1. Introduction

Due to a number of unique functional properties, organosilicate coatings (OSCs) are required for the nuclear power industry constructions [1,2,3], for instrumentation [4] and for anti-icing protection [5], including utilization in the Far North [6]. Among other applications, OSCs are used for the anticorrosion protection of bearing and enclosing constructions, reinforced concrete constructions and anchor bolts of railway contact network support foundations [7]. The problem with the OSCs behavior during installation and repair works, performed by arc welding, is still relevant when OSCs are used to protect metal constructions. One of the pioneers of the organosilicate materials technology, N.P. Kharitonov, demonstrated in his works [8,9] the possibility of steel construction repair using arc welding without preliminary removal of OSCs. It was considered that organosilicate materials melted and turned into a slag without forming inclusions in the weld at the steel melting temperature. Using OS-12-03, it was shown that the coating degradation width was 15–30 mm, the weld mechanical properties did not decrease, and there were no changes in the metal structure and composition (except magnesium content increasing) [7,9]. Due to the lack of newer works on the subject, there is a demand for further study employing modern research methods.

OSCs are based on toluene solutions of organosilicon and/or organic oligomers with a branched molecular structure, thereby making all these coatings heat-resistant [10]. The polymer base fillers are finely dispersed silicates, organic or inorganic pigments, and various target modifiers (curing agents, plasticizers, etc.) [2]. During the welding, before the organosilicon polymer degradation, OSCs have properties that are generally similar to paints and varnishes, and after polymer base degradation, OSCs behave like high-temperature inorganic composite materials [11], which are characterized by valuable special qualities: heat resistance, radiation resistance, etc. [12].

**Figure 1:**
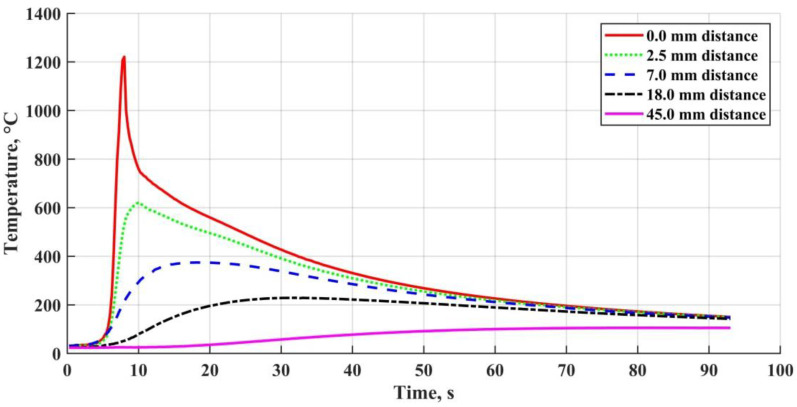
Welding thermal cycles at different distances from the weld boundary.

Earlier researchers had a somewhat different approach, and the OSCs transformation and decomposition processes at high temperatures were studied under mild heating conditions [9,13,14,15]. Regarding welding and its high temperature gradients, the previously defined reactions and their temperature range data [14,16] could only be used for the welded joint peripheral zone at the cooling stage. The experimentally obtained thermal cycles are shown in Figure 1 to demonstrate the heating rates and cooling time depending on the distance from the lateral weld boundary. The data were obtained by the thermal cycles acquiring using K-type thermocouples on 10 × 200 × 300 mm samples (presented below). From the decomposition onset temperature of the polymer component and until the steel melting temperature is reached, a number of physicochemical processes occur in OSCs, which may be classified into groups [17]:

(a) polyorganosiloxanes decomposition and formation of the crosslinks with the hydroxyl groups’ participation (or “siloxane structuring”) [18];
(b) dehydroxylation and other structural transformations of phyllosilicates [19];
(c) interaction of the polyorganosiloxanes breakdown products with the silicate and oxide components [14];
(d) new crystalline phases formation, the material transition to a ceramic-like state [20].

At a wide temperature range of 240–750 °C in the coating, the main processes taking place are film-former organic component depolymerisation, formation of crosslinks with the hydroxyl groups participation, and further decomposition with the various gaseous compounds release. For example, hydrogen, methane, benzene, water, formic acid, formaldehyde, carbon dioxide, and carbon monoxide, as well as three- and four-link methylcyclosiloxanes-hexamethylcyclotrisiloxane and octamethylcyclotetrasiloxane, are released during polydimethylphenylsiloxane thermooxidative decomposition [9]. Due to the presence of a carbon dioxide protective atmosphere, the polyorganosiloxanes’ “siloxane structuring” process occurs mainly in the peripheral areas at some distance from the weld. In these areas, there is currently no evidence of changes in the structure of the OSCs crystalline components, which is caused by the high individual heat resistance of the phyllosilicates (muscovite—1000 °C, talc—840–1050 °C [19,21]) and oxides in the absence of the contact with the reducing elements.

In the electric arc direct action zone and the surrounding area, even before the contact of the weld pool molten metal with the OSCs, the latter experience a “thermal shock”. The high heating rates (~10^2^ °C/s) cause the acceleration of the OSCs initial structure degradation. Decomposition and structuring ensue throughout the entire volume of the material; at temperatures above 500–720 °C, the formation of the new amorphous phases (including amorphous silica) is possible [9,22]. In this case, the formation of new phases common for mild heating is significantly slowed down or completely suppressed. [9,17,23]. Among the possible breakdown products of the OSCs organosilicon polymer base (regardless of its composition), hydrogen and water, dissociating in turn into atomic oxygen and hydrogen, have the main effect on the pore and nonmetallic inclusion formation [24]. Being in an atomic state, gases become chemically active, participating in the metallurgical processes with liquid metal, the result of which is the formation of volatile compounds and non-metallic inclusions in the weld. The main cause is the abrupt change in the solubility of gases during phase transitions in the metal. Dehydroxylation of the phyllosilicates and further decomposition of the water also increase the oxidation in the weld pool and the metal porosity. An additional effect on the porosity increase is exerted by the carbon oxidation in the steel with an oxygen excess [25].

Phyllosilicates’ high-rate thermal breakdown products appear to be an X-ray amorphous phase and poorly crystallized oxides; their amount among other breakdown products is considerably high [9,17,23]. For example, in the case of muscovite, SiO_2_ and Al_2_O_3_, in the case of talc, SiO_2_ and MgO, and, in the presence of both silicates, magnesium-aluminum spinel MgO·Al_2_O_3_. In this case, the latter, due to the lower wettability with liquid metal, will have a lower tendency to agglomerate in the weld, and aluminum oxide will have the greatest tendency (Al_2_O_3_ > MgO > MgO·Al_2_O_3_) [26]. SiO_2_ and TiO_2_, which interact most actively with the liquid steel, can be mostly removed into the slag.

In order to avoid inaccurate interpretation of the recommendations for OSCs application (VSN 436-82; RSN-40-81), it is expedient to clarify these recommendations using modern research methods. It is also important to obtain new data of the physicochemical processes occurring in coatings under the influence of a fast-acting and high-temperature processes of the arc welding. The aim of the present study was to investigate the distribution, composition, and morphology of the OSCs breakdown products in the form of non-metallic inclusions and also to determine the amount of diffusible hydrogen in the weld metal to clarify the recommendations for OSCs application.

## 2. Materials and Methods

Four different composition OSCs grades were selected for the study: weather-resistant, radiation-resistant OS-51-03 green and gray, anti-icing OS-56-22, and heat-resistant OS-82-01. The OSCs compositions are presented in Table 1. For the non-metallic inclusion distribution, composition, and morphology investigation experiment, the welding samples were produced using low-alloy mild steel sheets with the dimensions of 10 × 200 × 300 mm, with one 200 × 300 mm side coated. Both green and gray OS-51-03 and OS-56-22 coatings were cured by introducing a 0.5 wt. % 3-aminopropyltriethoxysilane hardener agent at the room temperature; composition OS-82-01 was cured in a furnace at a temperature of 250 °C with smooth heating for 3 h. The OSCs dry layer thickness (referred to below as thickness) was measured with a Konstanta K6 thickness meter. A welding bead was deposited on the coated sheet surface by the center along the 300 mm side using MAG (Metal Active Gas) welding in the carbon dioxide (CO_2_—100%) atmosphere using a Yaskawa Motoman MH24 robot and an EWM AlphaQ 552 power source. The welding parameters were: wire ESAB OK Autrod 12.51 with a diameter of 1.2 mm (d); welding current (I)—220 ± 10 A; arc voltage (U)—22 V; travel speed (V_ts_)—30 cm/min; wire feed rate (V_fr_)—6.5 m/min; torch was perpendicular to the substrate. Substrate and filler metal chemical compositions are presented in Table 2 (supplier data, incoming control by optical emission spectroscopy).

**Figure 2:**
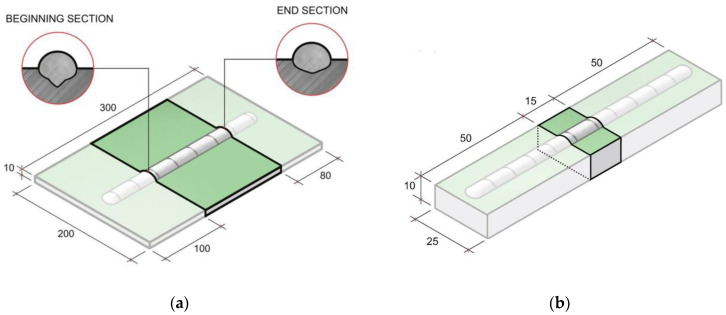
Samples for non-metallic inclusions investigation (**a**) and diffusible hydrogen amount determination (**b**).

After welding, at a distance of 100 mm from the beginning and 80 mm from the bead end, two sections were cut from each specimen for preparing the metallographic sections (Figure 2). The choice of the etchant was determined during the preparatory studies series. The etchant based on hydrochloric acid demonstrated the optimal ratio of microdefects detection and the noise spots absence. The etchants composition and purpose are shown in Table 3 [27]. The study of the non-metallic inclusion and micropore distribution was carried out using an optical microscope “Leica-DMI5000”. The average microdefect (non-metallic inclusion and less than 0.5 mm diameter micropore) diameter and their relative content in the weld metal were estimated using cross-section images with a 200-fold magnification using the automatic image processing algorithms in the Matlab software [28]. The three weld regions were investigated in the cross-section images: near the fusion boundary, the center and upper bead part. An image was converted into an 8-bit form (grayscale) by graphic editing software and then converted into a logical matrix using Matlab. The search algorithm for mircodefects with additional capabilities for screening out mircodefects by size, homogeneity, and eccentricity allowed for the distinguishing of non-metallic inclusions and micropores from noise spots with a high accuracy (about 0.025%). The full weld cross section relative porosity value was defined by estimating panoramic images at an x50 magnification by the same method. Porosity was defined as the pore area related to the entire cross section area of the test sample or the investigated region.

The question of the OSCs composition and thickness effect on the porosity and the welded metal quality was previously considered in detail in previous studies [29] and were taken out of this article scope.

The non-metallic inclusions elemental composition was determined by the energy-disparsive X-ray spectroscopy (EDS) in accordance with ISO 22309:2011 using a Tescan Mira3N scanning electron microscope (SEM) and an Oxford instruments X-max 80 Tescan Mira3N EDX detector.

The determination of diffusible hydrogen content in the deposited metal was carried out according to ISO 3690:2018 with the vacuum method [30], which is based on the hydrogen extraction from a specimen with a deposited bead: the hydrogen is extracted into a pre-vacuumed chamber connected to a manometer. The workpiece and the lead-out strips were produced from the mild steel sheet with 10 × 25 × 15 mm and 10 × 25 × 50 mm dimensions, respectively (Figure 2). The front side was coated. Before surfacing, the workpiece was clamped in a special copper device with water cooling. Robotic welding was carried out using ESAB OK Autrod 12.51 wire (d = 1.2 mm) in the mode: I = 200 ± 10 A; U = 20 ± 1 V; V_ts_ = 30 cm/min; V_fr_ = 6.0 m/min; contact-tip-to-work distance—10 mm. After deposition, the samples were cooled in ice water, separated from lead-out strips, and cleaned in toluene with a mechanical brush from the coating residues on the surface. Final samples cleaning was carried out by sequential washing in ethyl alcohol and acetone. The sample dried from the residual solvents was placed into the chamber. Air was pumped out to the operating pressure, which was controlled by a Setra 760 vacuum capacitance manometer.

## 3. Results and Discussion

### 3.1. Non-Metallic Inclusions Distribution

**Figure 3:**
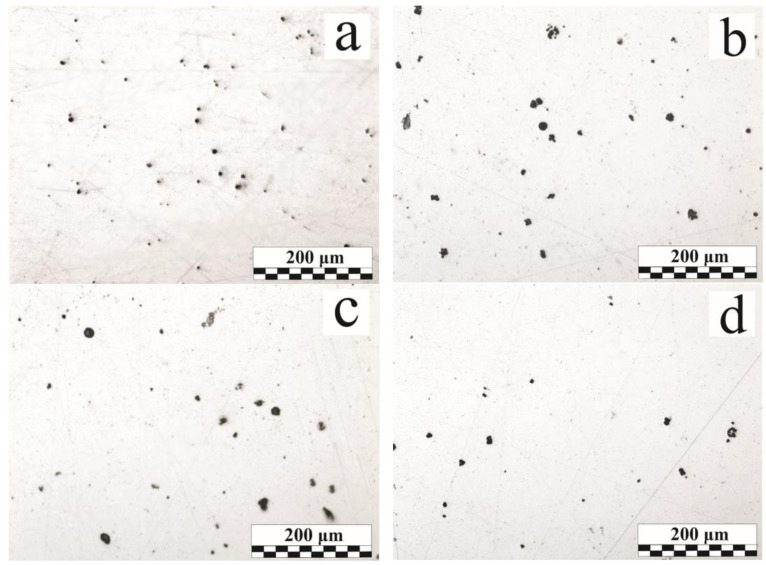
Weld cross-sections photographs: (**a**) bare sample, end; (**b**) OS-51-03 green, 95 microns, end; (**c**) OS-56-22, 185 microns, end; (**d**) OS-82-01, 160 microns, end.

The average diameter and the relative content of microdefects, as well as the relative weld metal porosity (considering the macropores), are given in Table 4. Figure 3 shows microsection images presenting the microdefect distribution in the weld. These images are the basis for the image processing analysis. Relative porosity values show that the dominant macropore influences the total weld metal porosity.

When using the above-mentioned etchants (Table 3), no homogeneous inclusions of TiO_2_, Cr_2_O_3_, or SiO_2_ were detected. The absence of homogeneous non-complex inclusions, which often are the higher oxides and do not dissolve in the liquid metal (unlike FeO) should indicate active interaction of OSCs breakdown products with components of liquid steel. In the case of refractory chromium oxide, stage reduction and further dissolution of chromium in liquid steel is most likely [31]. Silicon and titanium oxides are actively involved in the formation of complex non-metallic inclusions and the weld pool deoxidation, but the stage mechanics will strongly depend on local conditions—for example, the aluminum content [32,33].

The metal porosity depends on the distance from the weld beginning, and its increase is associated with the weld pool oversaturation with gases and is expressed in the sequential macrodefect accumulation during welding. Carbon monoxide or hydrogen may be the source of the pores; gases do not have time to be completely removed from the weld metal during the cooling before weld pool crystallization. Carbon monoxide is generated by the carbon oxidation by the atomic oxygen and the water vapor, along with the protective CO_2_ dissociation [34].

The relative non-metallic inclusion and micropore content have a slight tendency to increase along the weld from its beginning by an average of 20% and should be associated with an additional weld pool oxidation, as a result of which atomic oxygen actively interacts with the iron and the deoxidizing elements. FeO, which can dissolve in the liquid metal, poses a significant threat to the quality of the weld. This oxide has a melting point lower than the base metal, which leads to the interlayer formation along the grain boundaries and a decrease in the plastic properties of the weld metal [35]. OS-51-03 green data shows (Table 4) that the average microdefects size does not depend on the OSCs thickness; this parameter also does not change significantly with the distance from the weld beginning.

### 3.2. Non-Metallic Inclusions Composition and Morphology

**Figure 4:**
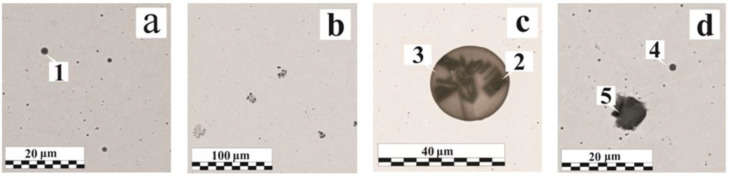
Nonmetallic inclusions in welds: (**a**) without coating; (**b**) with OS-51-03 green, 95 μm; (**c**,**d**) with OS-51-03 green, 115 μm.

In a typical MAG weld, the nonmetallic inclusions primarily depend on the composition of the base and filler materials, their purity, and the shielding gas quality. If the gas protection is not disrupted, the oxide inclusions of the FeO–MnO–SiO_2_–Al_2_O_3_ system are most often found in the low-alloy steel weld; the sulfide and oxosulfide inclusion presence is also possible [36,37]. Figure 4a shows the endogenous origin non-metallic inclusions distribution in the weld, and their typical compositions are presented in Table 5.

The presence of the OSCs breakdown refractory products in a supercooled liquid greatly facilitates the new phases emergence at the interface with the liquid due to a significant decrease in the nucleation process activation energy, in case the liquid wets the foreign phase surface. Such refractory compounds include oxides: Al_2_O_3_ (T_m_ = 2050 °C), MnO (T_m_ = 1780 °C), SiO_2_ (T_m_ = 1720 °C), TiO_2_ (T_m_ = 1870 °C), and others. Redox reactions can occur on the surface of these compounds, as a result of which the inclusion structure is noticeably more complicated due to the new phase formation. For example, titanium dioxide [38] is an active catalyst for heterogeneous crystallization in low-alloy steels.

In the welds performed on the 95–115 µm OS-51-03 green coating (Figure 4b), there are mainly oxide inclusions; the quantity of gas pores is small. In comparison with the reference sample (Figure 4a), the main changes were in the inclusions size rather than in their quantity. The inclusion size growth can be provided by one of two mechanisms: new phases precipitation intensification at the interface or the coagulation of small inclusions into larger ones. Examples of the first type are morphologically inhomogeneous (Figure 4c) and homogeneous (Figure 4d, Region No. 4) inclusions [39]. The stage-wise various phases separation is perfectly illustrated by a 25 μm diameter globular inclusion (Figure 4c). The central part contains more refractory aluminum oxide particles of an angular and irregular shape. During the crystallization process, silicon SiO_2_ and manganese MnO oxides, as well as a certain amount of the iron titanate FeO-Ti_2_O_3_, begin to precipitate on the aluminum oxide particle branched surface. The oxides’ relative content in the peripheral region is close to manganese anorthite MnO·Al_2_O_3_·2SiO_2_, which can be attributed to typical compounds formed during metallurgical processes [40].

**Figure 5:**
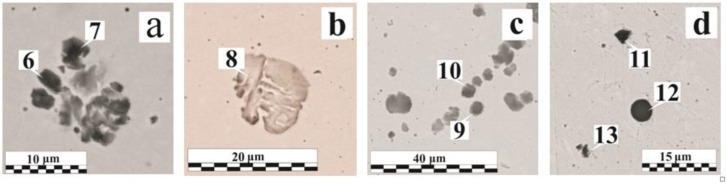
Nonmetallic inclusions in welds: (**a**) with OS-51-03 green, 95 μm; (**b**,**d**) with OS-51-03 green, 160 μm; (**c**) with OS-51-03 gray, 150 μm.

The second group is various chemical composition inclusions, enlarging after coalescence in the liquid phase mainly during the coagulation process. Due to their heterogeneity in their composition, inclusions can have different properties. In Figure 4d (Region No. 5), the inclusion has a low hardness and sufficient adhesion to the base metal; the inclusions shown in Figure 5a were shattered during the sample preparation due to the heterogeneity in the composition, and Figure 5b shows a mold of a previously contained non-metallic inclusion. The mold allows for the evaluation of the geometric shape of the inclusion. Irregular inclusions of 1–15 µm are mostly oxide compounds, sometimes with the presence of phosphates or sulfides (FeS, MnS).

**Figure 6:**
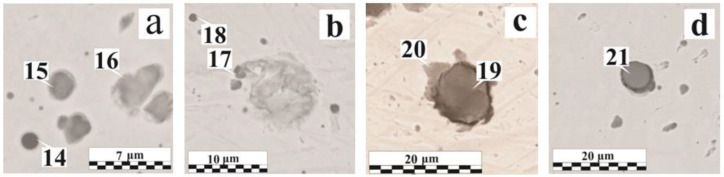
Nonmetallic inclusions in welds: (**a**,**d**) with OS-56-22, 185 μm; (**b**,**c**) with OS-82-01, 160 μm.

Figure 5c,d and Figure 6a show various defects accumulations explained by the formation under metastable equilibrium conditions in the weld metal area. Different types of inclusions can coexist: both large globular inclusions and irregular shapes with different morphology inclusions are observed in the cluster (Figure 5d). The irregular shape inclusion (Region No. 11) demonstrates the hercynite formation and is associated with the absence of the intense mixing in the liquid metal and the OSCs breakdown products’ low diffusion rate, which does not provide a uniform oxygen or deoxidizer distribution throughout the weld pool volume. The hercynite formation is also caused by a lower interfacial tension at the “FeO_x_·Al_2_O_3_—liquid steel” interface than that of a pure Al_2_O_3_. In the OS-56-22, despite the greater Mg amount (~4.3 wt. %) in the composition relative to Al (~0.4 wt. %), due to the better oxide wettability of the latter, a much greater presence of Al_2_O_3_ is observed in the non-metallic inclusions [26,41]. With a dissolved breakdown products concentration increase, nonmetallic inclusions serve as a surface for the gas pore formation (Figure 6b). In many pores, oxide inclusions were found, which emphasize an additional reason for the pore formation—the different thermal compression of inclusions and the steel matrix during cooling. The small pore shape, closely related by their origin to the inclusion presence, most often coincides with the inclusions shape [36].

In the initial component analysis, it was found that the talc contains impurities of calcium fluoride CaF_2_ and calcium phosphate Ca_3_(PO_4_)_2_ in amounts of up to 0.8 wt. %. Therefore, the fluorine presence in the non-metallic inclusions indicates phyllosilicates breakdown products. Despite such a low calcium content in the initial coating, calcium-containing inclusions (in most cases in the presence of phosphorus) were spotted in many samples. This may be due to the higher melting point of the hexagonal orthophosphate beta-modification relative to steel and the very poor solubility of calcium and its compounds in liquid steel [42] in the case of reduction, for example, with carbon.

3 Ca_3_(PO_4_)_2_ + 8 C = 3 Ca_3_P_2_ + 8 CO (900–1000 °C)

2 Ca_3_(PO_4_)_2_ + 10 C + 6 SiO_2_ = 6CaSiO_3_ + P_4_ + 10 CO (1000 °C)

No inclusions contained traces of volatile zinc oxide and barite breakdown products. Despite the significant chromium oxide presence in green coatings as a pigment, chromium is practically absent in inclusions. This may be due to the fact that, under welding conditions, the steel is cooled at such a rate that the chromium oxide phases of the inclusions are retained to room temperature in a metastable state, thereby fixing in the liquid and solid metal those components that form inclusions upon slow cooling.

Silicon oxide is poorly soluble in iron and floats into slag. Deoxidation with silicon is accompanied by formation reactions of more low-melting complex manganese and iron silicates, which transit better into slag.

Table 6 shows the typical non-metallic inclusions in the weld metal made according to OSCs.

### 3.3. Diffusible Hydrogen Amount in the Metal

**Figure 7:**
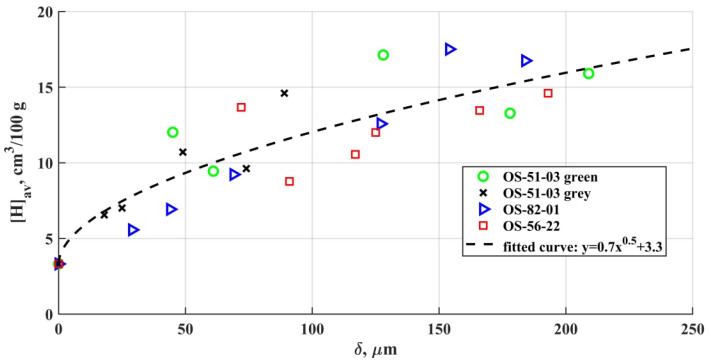
Relation between the diffusible hydrogen amount in the metal and OSCs thickness on the surface.

Figure 7 shows the diffusible hydrogen content value in the deposited weld metal for specimens coated with different OSCs. Even at a small OSCs thicknesses, an increase in the hydrogen content is observed, which indicates the coating breakdown in the arc zone with the formation of atomic hydrogen and its active dissolution in the weld pool metal. The amount of diffusible hydrogen in the deposited metal doubles even with a 25–50 µm OSCs thickness in comparison with a bare sample.

Establishing the relationship between the diffusible hydrogen amount and the hydrogen content in the coating is complicated by the need to consider the residual solvent content in the dry residue and some composition variability due to the use of components from different batches.

When a molten liquid metal interacts with a hydrogen-containing environment, hydrogen is absorbed by the surface until an equilibrium concentration with the environment is established [43]. The equilibrium concentration depends on the hydrogen pressure according to Sieverts’ law (square root law). The equilibrium distribution of hydrogen between the gas phase and the metal is achieved at:

[H_x_] = (KP_H2_)^1/2^

where [H_x_] is the concentration of hydrogen in the solution; P_H2_—hydrogen pressure in the gas phase; K—hydrogen distribution coefficient.

The amount of excess hydrogen released during the OSCs degradation does not exceed the limiting values of the hydrogen solubility in liquid steel. The obtained averaged dependences of the excess content of diffusible hydrogen (excess relative to uncoated samples) fully confirm the saturation of the weld pool with hydrogen in accordance with Sieverts’ law. The scatter of the obtained values has no tendency to increase with the growing thickness of the coating. Additionally, no correlation has been established between the deviation in the hydrogen content and such factors as the deposited metal mass decrease, the distortion of the area, and the shape of the weld surface. It should indicate insignificant changes in the rates of heat removal and convective mass transfer in the obtained samples [44].

The amount of diffusible hydrogen in the metal is not standardized for all steels. In general, for the mild steel utilized in this study, the increased content of diffusible hydrogen in the deposited metal is not an issue. At the same time, for the high-strength steels, as well as structures experiencing high loads, it is necessary to consider the cold cracking risks in accordance with the standards.

## 4. Conclusions

1. At the heating rates of ~10^2^ °C/s and with the temperatures up to 1200 °C, the OSCs initial structure is undergoing intense degradation. Hydrogen and oxygen, as the polymer base and hydrosilicates breakdown products, are the main elements that determine the defect formation in the weld. Hydrogen and carbon monoxide (formed as a result of a number of further reactions) are the main cause of pore formation in the metal. It was shown that the diffusible hydrogen amount rises along with the coating thickness. High metal oxidation results in the formation of nonmetallic inclusions of various morphologies and compositions, which are mostly based on the combinations of oxides: FeO, SiO_2_, MnO, Al_2_O_3_.
2. The total porosity increases from the weld beginning to its end as a result of the accumulation of dissolved OSCs breakdown products in the liquid metal and the emergence of the large macrodefects. The total area of micropores and non-metallic inclusions in comparison to macroporosity increases slightly from the weld beginning to its end. Nonmetallic inclusions and micropores are evenly distributed over the weld cross section.
3. The non-metallic inclusion size depends on the OSCs presence on the metal surface, and their composition also depends on the OSCs thickness. The inclusion size growth is provided by the intensification of the new phase precipitation at the interface or coagulation of small inclusions into larger ones. In the case of the intensification, the breakdown products of the heat-resistant phyllosilicates (Al, Mg), as well as Ca impurities, play an active role. The iron oxide inclusions mostly tend to grow due to coagulation. Inclusions of herzenite and alumina, fluorides, and phosphates are significantly rarer.
4. The amount of diffusible hydrogen in the deposited metal doubles even at a 25–50 µm OSCs thickness in comparison with a bare sample. For all four brands in the range of the examined thicknesses, the amount of diffusible hydrogen is closely related to the saturated hydrogen content, which correlated with the Sieverts’ law of the square root of the hydrogen fugacity.

## Figures and Tables

**Table 1:** Organosilicate coatings compositions and thicknesses.

Coating	Purpose	Organosilicon Binder	Pigments	Fillers	Polymer/(Fillers +Pigments) Ratio
OS-51-03 green	weather resistant, radiation resistant, deactivated	[(CH_3_)_2_SiO)_1.0_ (C_6_H_5_SiO_1.5_)_1.25_]_n_	TiO_2_ + Cr_2_O_3_	Mg_3_[Si_2_O_5_]_2_(OH)_2_ + KAl_2_(AlSi_3_O_10_) (OH)_2_	55/45
OS-51-03 grey			TiO_2_ + C_(black)_		
OS-56-22 (grey)	anti-icing	[(CH_3_)_2_SiO)_1.0_ (C_6_H_5_SiO_1.5_)_1.25_]_n_ + OHSi~ [(CH_3_)_2_SiO)]_n_ ~SiOH	ZnO + TiO_2_ + C_(black)_	Mg_3_[Si_2_O_5_]_2_(OH)_2_ + BaSO_4_ + KAl_2_(AlSi_3_O_10_) (OH)_2_	60/40
OS-82-01 (green)	heat-resistant	{[C_6_H_5_SiO_1.5_][CH_3_SiO_1.5_]_0.42_ [(CH_3_)_2_SiO]_1.33_}_n_ *	Cr_2_O_3_	KAl_2_(AlSi_3_O_10_) (OH)_2_	30/70

*—lacquer polymer is modified with organic polyester No. 315, which is synthesized from castor oil, diethylene glycol, maleic, and phthalic anhydrides.

**Table 2:** Substrate and filler metal chemical compositions.

Material	C	Si	Mn	Ni	S	P	Cr	N	Cu	As	Fe
	wt. %										
Substrate	0.14–0.22	0.15–0.3	0.4–0.6	≤0.3	≤0.05	≤0.04	≤0.3	≤0.008	≤0.3	≤0.08	bal.
ESAB OK Autrod 12.51	0.06–0.14	0.8–1.0	1.4–1.6	–	≤0.025	≤0.025	–	–	–	–	bal.

**Table 3:** Used etchants.

Etchant Composition	Etching Time	Main Etched Object	Detectable Inclusions
HCl—15 mL C_2_H_5_OH—85 mL	60 s	Cr_2_O_3_	FeS; MnS–FeS; Al_2_S_3_–FeS; FeO; MnO –SiO_2_; FeO –FeS; MnO–FeO–MnS–FeS; Al_2_S_3_–FeS–Al_2_O_3_–FeO; Cr_2_S_3_–FeS–Cr_2_O_3_–FeO
HF—50 mL H_2_O—50 mL	17 s	SiO_2_	FeS; MnS–FeS; Al_2_S_3_–FeS; Cr_2_S_3_–FeS; TiS_2_–FeS; FeO; CaO–SiO_2_; MnO–FeO; FeO–SiO_2_; MnO–SiO_2_; Al_2_S_3_–FeS–Al_2_O_3_–FeO
C_6_H_2_(NO_2_)_3_OH—2 g NaOH—25 g H_2_O—75 mL	150 s	TiO_2_	partially: FeS; MnS–FeS; Al_2_S_3_–FeS; MnO–FeO; FeO–FeS; MnO–FeO–MnS–FeS; completely darken: Cr_2_S_3_–FeS; FeO–TiO_2_–FeS–TiS_2_

**Table 4:** Micro- and macrodefects in the weld.

Sample	δ, μm	Average Microdefect Diameter, μm		Relative Microdefects Content, %		Relative Porosity (Including Macropores), %	
		Beginning	End	Beginning	End	Beginning	End
No coating	0	5.5 ± 1.5	4.9 ± 0.4	0.39 ± 0.23	0.27 ± 0.09	0.42	0.31
OS-51-03 green	95	5.6 ± 0.9	6.3 ± 0.4	0.55 ± 0.22	0.78 ± 0.31	0.61	0.69
	115	6.0 ± 0.7	5.8 ± 0.6	0.55 ± 0.21	0.59 ± 0.17	1.09	1.27
	160	5.0 ± 0.5	5.8 ± 0.5	0.36 ± 0.08	0.57 ± 0.08	0.36	14.27
OS-51-03 grey	150	4.2 ± 0.3	4.9 ± 0.6	0.29 ± 0.12	0.37 ± 0.22	0.63	8.18
OS-56-22	185	4.4 ± 0.7	4.3 ± 1.2	0.48 ± 0.11	0.52 ± 0.15	0.46	2.64
OS-82-01	160	3.6 ± 0.3	4.0 ± 0.8	0.37 ± 0.14	0.31 ± 0.11	0.54	1.49

**Table 5:** Non-metallic inclusions elemental composition in the weld metal.

Figure No.	Coating	δ, μm	Region No.	O	Fe	Mn	Si	Al	Ca	Cr	Mg	Ti	S	Others *
				Mass.%										
Figure 4a	without coating	0	1	22.9	54.5	12.3	9.9	–	–	–	–	–	0.4	–
Figure 4c	OS-51-03 green	95	2	45.2	1.5	3.4	1.5	48.0	–	–	–	0.5	–	–
			3	39.5	1.9	25.7	15.5	15.0	0.6	–	0.3	1.5	–	–
Figure 4d	OS-51-03 green	115	4	16.4	61.8	9.5	7.9	4.1	–	–	–	–	0.2	–
			5	26.9	65.9	0.9	2.2	–	1.2	0.3	0.4	–	0.3	P, Cu
Figure 5a	OS-51-03 green	115	6	16.0	81.0	1.0	0.9	–	0.3	–	–	–	–	P
			7	20.2	76.3	1.1	1.2	–	0.6	–	–	–	–	P
Figure 5b	OS-51-03 green	160	8	3.8	94.4	0.9	0.9	–	–	–	–	–	–	–
Figure 5c	OS-51-03 grey	150	9	7.0	88.1	0.8	2.7	0.3	0.6	–	–	–	0.2	P
			10	19.5	63.8	0.6	15.8	–	0.4	–	–	–	–	–
Figure 5d	OS-51-03 green	160	11	23.4	51.9	0.6	0.4	23.2	–	–	–	0.5	–	–
			12	46.6	2.6	4.1	15.4	9.0	17.4	–	2.1	2.7	0.2	–
			13	20.0	54.6	11.7	5.6	5.0	–	–	–	1.1	2.0	–
Figure 6a	OS-56-22	185	14	20.3	47.1	16.5	0.4	3.0	–	–	–	0.7	0.4	–
			15	11.9	75.8	4.5	5.9	1.2	0.3	–	–	–	–	P
			16	13.5	77.8	0.6	7.2	–	0.3	–	–	–	–	P
Figure 6b	OS-82-01	160	17	16.8	58.4	12.3	9.2	2.1	–	–	–	0.3	0.4	Na, Cl
			18	13.9	67.2	8.2	8.3	1.9	–	–	–	0.3	0.2	–
Figure 6c	OS-82-01	160	19	29.5	58.5	0.8	2.1	–	0.3	–	–	0.3	1.4	P
			20	10.0	86.9	0.7	1.2	–	–	–	–	–	0.2	P
Figure 6d	OS-56-22	185	21	39.1	3.0	24.8	19.5	9.8	0.2	–	–	0.9	0.5	F

*—due to the fact that the EDS method does not allow for the reliable quantification of the light elements mass fraction up to carbon inclusive, they are not taken into the table.

**Table 6:** Tensile properties of the studied joints.

Inclusion Type	Size, μm	Characteristic
Hercynite and Al oxides	3–20	Irregular triangular shape, can be separated or a part of complex inclusions
Fe-Mn-Si-Al (Ca or Mg) oxides	20–25	Usually large endogenous globular inclusions
Fe-Mn-Si oxides	2–3	Small globular inclusions, can be either separate or agglomerated into clusters
Fe-Si oxides	3–7	Relatively globular inclusions usually are presented in clusters
Fe oxides	12–15	Contain a small number of impurities, granular or globular
Fluorides, phosphates	2–4	Rare and often associated with the breakdown of heat-resistant phyllosilicates
